# *Toxocara canis*: Molecular basis of immune recognition and evasion

**DOI:** 10.1016/j.vetpar.2012.12.032

**Published:** 2013-04-15

**Authors:** Rick M. Maizels

**Affiliations:** Institute of Immunology and Infection Research, University of Edinburgh, Ashworth Laboratories, West Mains Road, Edinburgh EH9 3JT, United Kingdom

**Keywords:** Antibodies, Diagnosis, Larva migrans, Mucins, Surface coat

## Abstract

*Toxocara canis* has extraordinary abilities to survive for many years in the tissues of diverse vertebrate species, as well as to develop to maturity in the intestinal tract of its definitive canid host. Human disease is caused by larval stages invading musculature, brain and the eye, and immune mechanisms appear to be ineffective at eliminating the infection. Survival of *T. canis* larvae can be attributed to two molecular strategies evolved by the parasite. Firstly, it releases quantities of ‘excretory–secretory’ products which include lectins, mucins and enzymes that interact with and modulate host immunity. For example, one lectin (CTL-1) is very similar to mammalian lectins, required for tissue inflammation, suggesting that *T. canis* may interfere with leucocyte extravasation into infected sites. The second strategy is the elaboration of a specialised mucin-rich surface coat; this is loosely attached to the parasite epicuticle in a fashion that permits rapid escape when host antibodies and cells adhere, resulting in an inflammatory reaction around a newly vacated focus. The mucins have been characterised as bearing multiple glycan side-chains, consisting of a blood-group-like trisaccharide with one or two O-methylation modifications. Both the lectins and these trisaccharides are targeted by host antibodies, with anti-lectin antibodies showing particular diagnostic promise. Antibodies to the mono-methylated trisaccharide appear to be *T. canis*-specific, as this epitope is not found in the closely related *Toxocara cati*, but all other antigenic determinants are very similar between the two species. This distinction may be important in designing new and more accurate diagnostic tests. Further tools to control toxocariasis could also arise from understanding the molecular cues and steps involved in larval development. *In vitro*-cultivated larvae express high levels of four mRNAs that are translationally silenced, as the proteins they encode are not detectable in cultured larvae. However, these appear to be produced once the parasite has entered the mammalian host, as they are recognised by specific antibodies in infected patients. Elucidating the function of these genes, or analysing if micro-RNA translational silencing suppresses production of the proteins, may point towards new drug targets for tissue-phase parasites in humans.

## 1 Introduction

*Toxocara canis* is the most prevalent intestinal roundworm of dogs, foxes and other canid species, with zoonotic potential for human beings. In many temperate countries, toxocariasis is the most common helminth infection and causes a significant morbidity (Despommier, 2003; Hotez and Wilkins, 2009; Rubinsky-Elefant et al., 2010; Smith et al., 2009).

The nematode has many salient biological features, which contribute to its continuing presence in animal and human populations. Firstly, it is able to invade an extraordinarily wide range of hosts, from invertebrates and poultry (Galvin, 1964) through to mice and man (Strube et al., 2013); for all but the definitive (canid) species, these represent intermediate hosts which can, through predation, allow the parasite to reach its final definitive host species. Secondly, the larval stage is able to enter a long-term developmental arrest, which allows it to suspend life cycle progression whilst in a paratenic host, and to resume maturation to the adult stage only once reaching a canid species. Thirdly, even in female canids, developmental arrest occurs so that larvae can dwell in tissues until pregnancy occurs, and then migrate transplacentally or *via* colostrum to infect the foetal or newborn pup (Schnieder et al., 2011).

*T. canis* is highly prevalent in all canid populations that are not treated regularly with anthelmintics, and its infectivity to wild species renders elimination almost impossible. Furthermore, the arrested state has remarkable longevity: in experimentally infected monkeys for example, larvae remained viable in the tissues for 9 years and were able to infect mice on transfer (Beaver, 1962).

Within the paratenic host, *T. canis* larvae can migrate widely including the liver, musculature and the central nervous system, causing the well-characterised syndrome of visceral larva migrans (VLM) (Beaver et al., 1952; Carvalho and Rocha, 2011; Schantz, 1989). The propensity to invade the brain and the eye has given rise to particular concern in the human population, with ocular toxocariasis (OT) a recognised syndrome (Good et al., 2004), and neurotoxocariasis (NT) inferred from cognitive deficits, higher prevalence among epilepsy cases (Quattrocchi et al., 2012), and finding larvae in post-mortem brain samples (Hill et al., 1985). Current options for treatment of humans infected with tissue-dwelling larvae are of uncertain efficacy because of the covert nature of the infection and the incomplete resolution of symptoms (Othman, 2012; Wiśniewska-Ligier et al., 2012).

## 2 Immune recognition

Pathogens are recognised initially by the innate immune system reacting to intrinsically foreign molecular signatures associated with xenogeneic species, such as bacterial lipopolysaccharide, unmethylated DNA and fungal carbohydrates. Such signals are essential for innate immune sentinel cells, such as dendritic cells, to react to the presence of infectious organisms and to initiate pathogen-specific responses from the adaptive immune system. Currently, few such “pathogen-associated molecular patterns” (PAMPs) have been defined for any helminth parasite, but their existence in *T. canis* can be surmised by the strong adaptive immune response that occurs in this infection. The major feature of this adaptive immune response is production of *T. canis*-specific antibodies, associated with CD4+ T-helper type 2 cell (Th2) activity (Del Prete et al., 1991).

Such a Th2 response is characterised by release of a specific subset of mediators, in particular the type 2 cytokines IL-4, -5, -10 and -13, during infection. Subsequently, IL-4 promotes B cell differentiation and antibody class-switching, while IL-5 drives the differentiation of eosinophils, a marked feature of human *Toxocara* spp. infection (Beaver et al., 1952). T cell responses to *T. canis* in human beings are clearly of the Th2 type, and are stimulated by the “excretory–secretory” (ES) antigens of the parasite (Del Prete et al., 1991). However, no individual T cell specific antigen or epitope has yet been defined from *T. canis*.

Recently, the distinction between innate and adaptive arms of immunity has become much more blurred with identification of innate lymphoid cells, which can rapidly produce Type-2 cytokines (Neill et al., 2010) without requiring activation by dendritic cells (Smith et al., 2012). Indeed, it has been known for some time that some innate populations of cells can participate in the “Th2 response” to *T. canis*, for example non-B-non-T cells produce IL-5, even in T cell-deficient nude mice infected with the parasite (Takamoto et al., 1995). Moreover, eosinophilia in infected mice shows a biphasic response with early (day 10) and late (day 21) peaks; while the later peak is absent in CD4+ T cell-deficient mice, the earlier one is intact, indicating that it is generated by the innate rather than the adaptive Type-2 response (Takamoto et al., 1998).

Generation of specific antibodies provides the most definitive evidence for infection and is the basis for all diagnostic tests for toxocariasis; with ELISA and Western blot reactivity to *T. canis* ES (TES) antigens generally employed. However, the diagnostic field is still evolving with respect to the optimal target antigens that should be used, and more critically to the interpretation of antibody reactivity to an infection which is frequently covert and in which symptoms do not necessarily correlate with infection intensity or antibody titre (Smith et al., 2009).

There are now extensive data on the seroprevalence of anti-TES antibodies in human populations around the world, using the established ELISA. In older studies, seropositivity in Europe ranged from 3 to 7% in adults and 7 to 23% in children (Gillespie et al., 1993), while in the USA 13.9% of those over 6 years of age were antibody-positive (Won et al., 2008). Remarkably, seroprevalences exceeded 50% in tropical settings such as the Caribbean (Bundy et al., 1987; Magnaval et al., 1994). More recent studies similarly report seroprevalences of 14.5% in Polish teenagers (Jarosz et al., 2010), 13% in Turkish children (Doğan et al., 2007) and 9% in Iranian under-10s (Fallah et al., 2007), rising to 27% in Brazilian Amazonia (Rubinsky-Elefant et al., 2008) and 45–82% in different rural districts of Sulawesi, Indonesia (Hayashi et al., 2005). Hence widespread toxocariasis remains of great concern in most parts of the world.

The question of which serum antibody isotypes are most relevant still remains to be explored. Most human infections generate antibodies of the IgG1 subclass, with significant levels of both IgM and IgE (Smith, 1993). The IgG4 isotype, which can be predominant in other, more intense, tissue helminth infections such as filariasis and schistosomiasis (Maizels et al., 1995) is less prominent, although reportedly more evident in active cases of visceral larva migrans (Obwaller et al., 1998). An unresolved issue is whether the expression of IgE correlates with active infection and/or invasion of certain tissue sites in the host, or possibly mediates only bystander allergic-type reactions such as infection-related rashes (Magnaval et al., 2006). Related to this question is whether particular isotypes are functionally important in tissue immunity, for example by trapping larvae or activating Fc-dependent protection mechanisms.

Recent analyses of differential expression profiles of anti-*Toxocara* spp. antibody isotypes, studying children receiving thiabendazole therapy, found that specific IgE and eosinophilia declined within the first year, with IgA and IgG4 falling at later times (Elefant et al., 2006). Such information may prove useful in addressing the need for new immunological markers that could be used to chart the success of therapy in drug-treated patients.

## 3 Immunity in the definitive host

Although almost all immunological studies on *T. canis* have centred a round the tissue-migrating larvae, the immune response of the final canid host to intestinal adult worms is also of central importance. A long-term aim of research in this area is to develop an effective vaccine against canine toxocariasis, which ideally would build immunity to both tissue- and intestinal stages of the parasite.

Intestinal immunity to ascarid worms is poorly understood; taking a comparative approach, based on animal models of taxonomically distant nematodes (*e.g. Nippostrongylus brasiliensis*, *Trichuris muris*), it would be predicted that Th2-dependent mechanisms act to expel the parasites, but the cell types and molecular mediators participating in expulsion are as yet unknown. Notably, it has been reported that Foxp3+ regulatory T cells are more numerous in dogs with intestinal nematode infections (Junginger et al., 2012), suggesting that mucosal tolerance is involved in persistence of infection. Thus, interventional strategies to neutralise Tregs could promote protective immunity, as has been reported in some animal models of helminth infection (Taylor et al., 2007).

Immunity to the larval stage is similarly poorly understood, although important not only to the definitive canid hosts, but also in the human setting. To date, attempts to generate protective anti-larval immunity in laboratory rodents have had mixed results. While an early report noted that immunisation with somatic extracts of eggs or adult worms resulted in approximately half the number of surviving challenge larvae (Izzat and Olson, 1970), susceptibility to larval infection was unaltered in mice immunised with UV-irradiated eggs or larval ES antigens (Abo-Shehada et al., 1991). Likewise, a more recent study found no evidence of protection by administering a second challenge of larvae to previously infected mice, other than they were more likely to sequester in the brain (Kolbeková et al., 2011). A more systematic approach, comparing immunisation and adjuvant regimes and following more closely the resultant immune response, would help clarify this question.

## 4 *Toxocara* spp. and allergy

Many, but not all, studies have linked human *Toxocara* spp. infection with exacerbation of allergic disease (reviewed by Pinelli and Aranzamendi (2012)). Children with *T. canis* seroreactivity have been reported to show more pronounced IgE and eosinophilia than seronegative individuals as well as greater propensity for airway allergic symptoms such as asthma (Buijs et al., 1997) and allergic rhinitis (Yariktas et al., 2007). Mice infected with *T. canis* developed compromised lung function for up to 60 days, associated with bronchioalveolar eosinophilia and serum IgE production (Pinelli et al., 2005). Moreover, using a protocol in mice which induces an airway allergic response to the model antigen ovalbumin, *T. canis* infection prior to ovalbumin challenge was found to exacerbate lung pathology (Pinelli et al., 2008).

On a broader scale, infection with *T. canis* might predispose human beings towards development of asthma (Cooper, 2008). Such pro-allergic effects seemingly run counter to a recent interpretation of the “hygiene hypothesis”, which suggests that parasitic helminths dampen allergies and other immunopathologies through their general immune suppressive properties (Maizels, 2005). However, it is important to recognise that *T. canis* is adapted to its definitive host rather than to intermediate or accidental hosts such as the human, and in the maladapted setting, the host may react more vigorously and pathologically.

## 5 Evasion of the immune response

Long-term survival of parasitic helminths in the host is an impressive feat in the face of the well-armed immune response, which mobilises multiple cell types and molecular mediators, including high-affinity antibodies, to attack invading organisms. *T. canis* larvae invade from the host intestinal tract and disseminate throughout the tissues of the body, remaining in an extracellular state, generating little inflammatory reaction at the site of infection. Therefore, it is implicit that *T. canis* is able to disable host effector mechanisms in an extremely effective fashion.

One cell type, associated with immunity to helminths in the tissues, is the eosinophil (Klion and Nutman, 2004; Maizels and Balic, 2004), which is able to exgorge noxious products such as major basic protein, as well as generate superoxides (through eosinophil peroxidase) and other damaging free radicals. Although eosinophilia is certainly a prominent feature of toxocariasis, as indeed in many other helminth infections, it appears that *T. canis* is largely resistant to attack by this cell type. In mice over-expressing an IL-5 transgene (IL-5T) and with resultant hypereosinophilia, *T. canis* larvae are unharmed, although an unrelated helminth *N. brasiliensis* is eliminated (Dent et al., 1999). Notably, when *N. brasiliensis* is introduced to IL-5T mice in the presence of *T. canis* ES antigens (see below), their survival is greatly enhanced (Giacomin et al., 2008). Conversely, in IL-5-deficient mice there is no change in larval survival, although pathology in the lung is diminished compared to wild-type (IL-5-sufficient) controls (Takamoto et al., 1997).

## 6 Shedding of the surface coat

The inability of eosinophils to kill *T. canis* was observed *in vitro*, in experiments in which cells and larvae are co-incubated. While in the presence of specific antibodies, eosinophils rapidly adhered to the parasite surface, but within 24 h the larvae had escaped from surrounding cells (Fattah et al., 1986), leaving the eosinophils still attached to material which had clearly become detached from the surface (Badley et al., 1987a).

These observations of shedding cells bound to the parasite surface have an interesting link to electron microscopic observations of a labile surface coat from the larval parasite, distal to and separated from the nematode cuticle. When larvae are treated with a lipophilic stain, a “fuzzy coat” can be visualised, 10–20 nm thick and lying approximately the same distance from the cuticle surface, while following each cuticle annulation and fold (Page et al., 1992b). The fuzzy coat can be stained with polycationic ligands such as ferritin, indicating that it bears an overall negative charge. Notably, the coat is lost within 2 h of co-incubating larvae with antibodies reactive to the surface, elucidating the physical basis of the original report of antibody shedding by *T. canis* larvae *in vitro* (Smith et al., 1981).

While the first observations of antibody shedding employed polyclonal sera, later studies confirmed this with monoclonal antibody (MAb) reagents. In particular, two MAbs designated Tcn-2 and Tcn-8, which bind strongly to live larvae, and which are rapidly shed, were also demonstrated by immuno-electron microscopy to bind the surface coat (Page et al., 1992b). Interestingly, the same antibodies were used to stain sections of larvae, and identified two distinct secretory bodies within the larva, the oesophageal gland opening into the buccal cavity, and the mid-body secretory column which is a ducted secretory pore (Page et al., 1992a).

The Tcn-2 and Tcn-8 monoclonal antibodies which bind the surface coat, recognise related but distinct trisaccharide glycan side-chains (Schabussova et al., 2007), which are expressed on the family of mucin polypeptides, principally MUC-1, -2 and -3 collectively termed TES-120 (Loukas et al., 2000b). The coat and the secretory column are also bound by the N-acetylglucosamine-specific lectin, Wheat Germ Agglutinin (Page et al., 1992c). While the fuzzy coat therefore seems likely to represent a mucin- and glycan-rich assembly, it is not yet determined if the mucins are further covalently modified in any way, or linked to other molecular components, in order to form a stable coat structure.

## 7 TES antigens and immune modulation by TES antigens

While the ability of larvae to escape antibody and cellular attack is unusual among helminth parasites, a more general feature is modulation of the systemic immune response to infection. In *T. canis* infected mice, the pro-inflammatory responses of macrophages are dampened (Kuroda et al., 2001), while susceptibility to a sub-lethal dose of Japanese encephalitis virus is increased (Gupta and Pavri, 1987). Recent work has reported an expansion of the suppressive T cell subset of Foxp3+ regulatory T cells in murine toxocariasis (Othman et al., 2011), which may underpin some of these *in vivo* effects.

It is likely that both local and systemic effects of parasite infection on host immunity are mediated by secreted products, such as the TES antigens. As mentioned previously, TES can inhibit the protective effects of eosinophils (Giacomin et al., 2008) and is able to induce recruitment of both eosinophils (Sugane and Oshima, 1984) and alternatively activated macrophages (Allen and MacDonald, 1998). However, TES is a complex mixture of differing molecular components, which require further characterisation if the specific immunomodulatory effects of the parasite are to be understood.

de Savigny (1975) first reported that *T. canis* larvae show remarkable longevity *in vitro*, allowing long-term collection of ES antigens over many months. As detailed below, such “TES” proved to be extremely useful for diagnostic purposes. Within TES, the molecular components were first described by their molecular weight on SDS-PAGE analysis, with major bands evident at 26, 32, 45, 55, 70, 120 and 400 kDa (Maizels et al., 1984); even at this stage the extensive overlap between surface proteins and those in TES was clear.

Subsequent studies of TES used 1- and 2-dimensional SDS-PAGE to more precisely classify components (Badley et al., 1987b; Meghji and Maizels, 1986), together with techniques such as biosynthetic labelling with radioactive amino acids (Page and Maizels, 1992; Sugane et al., 1985). A high degree of glycosylation was observed, amounting to ∼40% of TES by weight (Meghji and Maizels, 1986). Histological sections of liver from infected mice also showed that TES is released from larvae *in vivo* (Parsons et al., 1986).

Over subsequent years, a combination of peptide sequencing (Loukas et al., 1999b), monoclonal antibody binding (Bowman et al., 1993; Maizels et al., 1987) and recombinant DNA techniques (Gems et al., 1995; Gems and Maizels, 1996; Maizels et al., 2000) has characterised three sets of TES proteins and glycoproteins (TES-26, -32/70 and -120) while the other components remain to be fully characterised.

### 7.1 TES-26: Tc-PEB-1

TES-26 is a homologue of mammalian phosphatidylethanolamine (PE)-binding protein and was renamed Tc-PEB-1. It was identified initially from an abundant mRNA bearing at its 5′ end the conserved a nematode 22-nt spliced leader (SL1) sequence found previously in *Caenorhabditis elegans* (Krause and Hirsh, 1987). While the *T. canis* PEB protein is similar to homologues from other species in functionally binding PE, its structure differs markedly in including a 72-aa N-terminal extension, containing a tandemly repeated six-cysteine motif homologous to the potassium channel toxin of the sea anemone, *Stocihactis helianthus* (Gems et al., 1995). The *S. helianthus* K-toxin (ShKT) domain figures prominently in a number of other secreted proteins not only from *Toxocara* spp., but also from other nematode parasites, although as yet has no known functional role.

### 7.2 TES-32 and -70: C-type lectins (CTLs)

TES-32 (Loukas et al., 1999b) and TES-70 (Loukas et al., 2000a) were isolated by matching tryptic peptide sequences from gel-purified proteins to an EST database of larval mRNA sequences (Tetteh et al., 1999). Both are members of the C-type lectin family; hence TES-32 was reassigned at Tc-CTL-1. This is homologous to mammalian mannose-binding protein, and modelling studies which indicated that Tc-CTL-1 does indeed bind mannose residues were experimentally confirmed (Loukas et al., 1999b). Within the EST database were two related sequences with minor amino acid variants which have been designated Tc-CTL-2 and -3. A near-identical sequence was later described by an independent group as a proteoglycan core protein (Yamasaki et al., 1998).

Tc-CTL-1 is also one of the major larval surface proteins, and monoclonal antibody localisation has shown that it is expressed in the cuticle of the parasite (Page et al., 1992a). Whether the cuticular form bears any modification, and/or is secreted directly from the cuticle into the larval environment, remains to be determined. Tc-CTL-1 also appears to be well-conserved within the *Toxocara* genus; the anti-Tc-CTL-1 Mab Tcn-3 reacts with proteins in both *Toxocara cati* (Kennedy et al., 1987) and *Toxocara vitulorum* (Page et al., 1991), albeit of differing molecular weights. In addition, there is significant cross-reactivity of Tc-CTL-1 with antibodies generated to the raccoon ascarid *Baylisascaris procyonis* (Boyce et al., 1988). However, there is no cross-reaction with ES from *Toxascaris leonina* (Page et al., 1991) and mice infected with *Ascaris suum* do not develop serum antibodies to Tc-CTL-1.

TES-70 is a further C-type lectin, now referred to as Tc-CTL-4, which has been shown to bind to canine cells *in vitro*. Although the mammalian ligand has yet to be identified, this is the first instance of a TES product directly targeting host receptors (Loukas et al., 2000a). Monoclonal antibodies to Tc-CTL-4 also react with products in ES of other *Toxocara* species, indicating that secretion of C-type lectins might be a shared hallmark of this genus (Page et al., 1991).

### 7.3 TES-120: mucins

As with TES-26, the original isolation of cDNA clones corresponding to TES-120 antigens was facilitated by the presence of the SL1 trans-spliced leader sequence. The conserved sequence of the SL1 permitted full-length cloning, without the requirement for a cDNA library or development of specific probes (Gems and Maizels, 1996). The most highly expressed gene encoded a mucin sequence, designated MUC-1. Further studies established that *T. canis* larvae express a set of mucin-like glycoproteins with extensive Serine-Threonine rich domains which act as sites of O-linked glycosylation, forming a surface “fuzzy coat” described previously (Page et al., 1992b). The principal mucins are MUC-1 (Gems and Maizels, 1996), -2 and -3 (Loukas et al., 2000b) with less abundant components MUC-4 and -5 (Doedens et al., 2001), differing in structure and the relative proportions of Serine or Threonine residues (Loukas et al., 2000b). Most strikingly, all contain tandemly repeated ShKT domains at either or both of the N- and C-terminal regions, flanking the central mucin core.

## 8 Glycans of *T. canis*

Initial chemical analysis of TES revealed a high content of galactose and N-acetylgalactosamine, amounting to ∼40% by total weight of the secreted products (Meghji and Maizels, 1986). However, there is only a limited degree of N-glycoslyation, of CTL-1 and -4, as revealed by molecular weight shifts following N-glycanase digestion of total TES products. In contrast, O-linked sugars represent the majority of the released carbohydrates, mostly linked to the mucin families (MUC-1 to -5).

Mass spectrometry of the O-linked sugars determined them to be two related trisaccharides, 2-O-Me-Fucα1-2(4-O-Me)Galβ1-3-GalNAc, and 2-O-Me-Fucα1-2Galβ1-3GalNAc, differing only in whether the central galactose sugar bears an O-methyl side chain (Khoo et al., 1991). Notably, the non-O-methylated form of this trisaccharide is identical to the human blood group H antigen, providing a structural explanation for the expression of blood group specificities by *T. canis* (Smith et al., 1983). Chemical synthesis of the *Toxocara* spp.-specific glycan structures (Amer et al., 2001) provided material for ELISA with monoclonal antibodies and human sera (Schabussova et al., 2007). This confirmed that the species-specific Mab, Tcn-2, recognises the mono-methylated trisaccharide, while the shared epitope present in other *Toxocara* species is the dimethylated structure (Schabussova et al., 2007).

Subsequently, synthesis of the glycan Fucα1-2Galβ1-3GalNAc, omitting methylation of either the terminal fucose or the intermediate galactose, was used to demonstrate loss of antibody binding, formally confirming that the Me-Gal residue is essential for this reaction (Koizumi et al., 2012). In addition, analysis of the N-linked glycan species in TES antigens has been undertaken, identifying a dominant and relatively simple biantennary Man2Man-GlcNAc-GlcNAc side chain (Khoo et al., 1993).

## 9 Immuno-diagnosis

The original reports from De Savigny (de Savigny and Tizard, 1977; de Savigny et al., 1979) provided a breakthrough for immuno-diagnosis of toxocariasis, as the sensitivity and specificity of TES in assays such as ELISA and Western Blot proved to be far superior to use of whole worm extracts (Van Knapen et al., 1983). Indeed, the use of TES has been successfully translated into a commercial diagnostic kit (Jacquier et al., 1991). Nevertheless, the sensitivity and specificity of TES-based immunodiagnostic tests vary around the 90% level, and there are concerns that the presence of blood group-like or other cross-reactive carbohydrates could be responsible for a number of false-positive results (Smith et al., 1984, 2009). The blood group-like antigens are more abundant on higher molecular-weight TES components (TES-120 and TES-400), and therefore a Western blotting procedure to assay human antibodies to the lower molecular weight TES components (TES-26 and TES-32) has also been developed (Magnaval et al., 1991, 2002).

Recombinant proteins are now being developed for detection of specific antibodies for toxocariasis. The TES-32 protein (which corresponds to the C-type lectin CTL-1 (Loukas et al., 1999b)) was shown to be detected by serum antibodies from all 9 toxocariasis patients tested. In addition, sera from animals carrying *T. cati* infection were also reactive, reflecting the cross-reactivity of this protein between *Toxocara* species (Kennedy et al., 1987). In a standard ELISA assay, some 43% of sera from other helminth infections cross-reacted with whole TES, but this was reduced to 2% when tested against recombinant CTL-1 adsorbed at an optimal antigen concentration (Yamasaki et al., 2000). In a subsequent evaluation, sera from 26/215 (12%) of children from Pernambuco, Brazil, recognised Tc-CTL-1 (TES-32) (De Andrade Lima Coêlho et al., 2005). It should be noted that in both studies, recombinant Tc-CTL-1 was produced as inclusion bodies in bacteria and required 8 M urea solubilisation, indicating that a correctly folded version of the same antigen may show increased immunoreactivity as this would detect conformational as well as linear epitopes.

Most diagnostic serological tests measure total IgG antibodies. In a comparative study to test an IgG4-specific assay, it was noted that sensitivity was markedly reduced (as many patients do not mount an IgG4 response) but specificity was greatly enhanced compared to measuring total IgG (Noordin et al., 2005). These authors went on to develop an IgG4-specific diagnostic assay employing three recombinant antigens (Tc-PEB-1 or TES-26, Tc-CTL-1 or TES-32 also named TES-30USM, and Tc-MUC-1 or TES120). While none of the antigens individually showed 100% specificity, a combination of the three was able to detect all samples serologically reactive to native TES, and showed cross-reactivity with only 2–4% of serum samples from other infections (Mohamad et al., 2009).

As well as recombinant proteins, the specific glycan structures associated with *T. canis* also hold promise for immuno-diagnosis. It was found that sera from human toxocariasis patients reacted to the di-O-methylated structure 2-O-Me-Fucα1-2(4-O-Me)Galβ1-3-GalNAc, although no antibodies were detected to the species-specific monomethylated form Schabussova et al. (2007). As is often the case with glycan epitopes, human antibodies were restricted to the IgM and IgG2 isotypes.

Using polyclonal antibodies against TES in a sandwich ELISA, circulating parasite antigens can be detected in the serum of infected dogs, particularly pups (Matsumura et al., 1984), and in serum of children with suspected infection (Luo et al., 1999). Similarly, circulating antigen could be found in both human and experimental mouse serum samples, with the monoclonal antibody Tcn-2 which recognises a species-specific repetitive epitope that facilitates detection (Gillespie et al., 1993; Robertson et al., 1988).

## 10 Genomics, transcriptomics and other molecular analyses

Despite the importance of *T. canis* in both veterinary and zoonotic contexts, there is relatively little information available at the genomic and transcriptomic levels. Currently, the parasite genome has yet to be sequenced. However, the 14,162-bp mitochondrial sequence has been published (Jex et al., 2008), and the 273-Mb genome sequence of the related parasite *A. suum* was recently reported (Jex et al., 2011).

Similarly, the *T. canis* transcriptome is relatively under-explored (Gasser, 2013). Over 10 years ago a small-scale EST project on larval cDNAs was able to identify 128 distinct genes and despite its limited scope, this study characterised most of the major secreted antigens and a number of novel gene families, many of which are listed in Table 1 (Tetteh et al., 1999). A further 1975 gene clusters were assembled from ∼5000 Sanger sequencing reads and can be searched at http://nematode.net. More recently, a female adult cDNA sequencing effort yielded 79 genes, including 2 C-type lectins and an As16 homologue (Zhou et al., 2011). However, most of the identified genes have yet to be annotated or deposited in public databases, with NCBI for example holding only 28 distinct non-mitochondrial/ribosomal protein gene sequences at the time of writing. Much more ambitious datasets are required to establish the protein repertoire of *T. canis* and to identify patterns of stage-specific protein expression in the parasite life cycle.

In the last few years, a new class of biological regulatory molecules has been defined with the discovery of small (∼22-nucleotide) RNA species which bind to and inhibit translation of target mRNAs and mediate their degradation within the cell. Most documented cases of miRNA binding are to the 3′ untranslated region (3′ UTR) downstream of the translational stop codon. Abundant miRNAs have been described in several nematode species (Chen et al., 2011; Poole et al., 2010; Winter et al., 2012), most notably the free-living worm *C. elegans*, which provides well-characterised conserved sequences such as *let-7* (Ambros et al., 2003; Hammell et al., 2009). Although no miRNAs have yet been described in *T. canis*, it had been noted that 4 abundant novel transcript (ANT) genes from the larval stage shared highly similar 3′ UTR sequences despite dissimilar coding sequences (Tetteh et al., 1999). Interestingly, in the larval parasite, no evidence of proteins corresponding to the ANT genes was found, indicating that either gene transcription or mRNA translation was suppressed within the arrested stage. Within the 3′ UTR tracts of each transcript were motifs similar to those targeted in *C. elegans* by *let-7*, and when the 3′ UTR of a *T. canis* ANT gene was inserted downstream of a GFP construct in transgenic *C. elegans*, suppression of gene expression was achieved (Callister et al., 2008). Thus, although the involvement of canonical miRNA mechanisms in control of larval gene expression has yet to be directly demonstrated, these studies strongly suggest that this is the case.

## 11 Future prospects and priorities

Expanding our understanding of immune recognition and evasion by *T. canis* is a priority if we are to appropriately manage both human and veterinary disease. Molecular studies to date have identified key products which are already being used in new diagnostic assays, and doubtless further refinement will lead to the replacement of native TES as the principal diagnostic reagent. Beyond this very necessary innovation, two general and interrelated areas of research need to be addressed: by what molecular means does *T. canis* suppress host immunity, and how can we intervene to provide immunological protection in either or both the definitive and paratenic host? These questions should spotlight the highly evolved state of immune evasion by *T. canis*, with every probability of defining new immunomodulatory molecules. In this way, future work can pave the way for rational immunological tools, most importantly vaccines for use in canid species, to mimimise, if not altogether eradicate, this important infection.

## Conflict of interest

None.

## Figures and Tables

**Table 1:** Defined proteins of *Toxocara canis*.

Abbreviation	Name	TES component if applicable	Accession number(s)	Length (incl signal sequence if present)	Notes	Reference
Tc-ANT-3	Abundant novel transcript-3		EU792508; ACF19852	271 aa		Callister et al. (2008)
Tc-ANT-5	Abundant novel transcript-5		EU792509; ACF19853	489 aa		Callister et al. (2008)
Tc-ANT-30	Abundant novel transcript-30		EU792510; ACF19854	843 aa		Callister et al. (2008)
Tc-ANT-34	Abundant novel transcript-34		EU792511; ACF19855	608 aa		Callister et al. (2008)
Tc-AQP-1	Aquaporin		AAC32826	310 aa		Loukas et al. (1999a)
Tc-ARK-1	Arginine kinase		ABK76312; AFJ95132			Unpublished
Tc-CPL-1	Cathepsin L-like cysteine protease		AAC48340	360 aa		Loukas et al. (1998)
Tc-CPZ-1	Cathepsin Z-like cysteine protease		AF143817	307 aa		Falcone et al. (2000)
Tc-CTL-1	C-type lectin-1	TES-32	AF041023	219 aa	Binds mannose	Loukas et al. (1999b)
	Proteoglycan core protein		AB009305	219 aa	Differs by 3 aa from above	Yamasaki et al. (1998)
Tc-CTL-2	C-type lectin-2		–	219 aa	17% aa divergence from CTL-1	Tetteh et al. (1999)
Tc-CTL-3	C-type lectin-3		–	220 aa	13% aa divergence from CTL-1	Tetteh et al. (1999)
Tc-CTL-4	C-type lectin-4	TES-70	AF126830	288 aa	Binds canine cell surface	Loukas et al. (2000a)
Tc-GLB-1	Pseudocoelomic globin		AAL56430; AAL58703; AAL58704	171 aa	Minor sequence variants	Unpublished
Tc-GLB-2	Intracellular globin		AAL56428; AAL56429	153 aa	∼50% identity to GLB-1	Unpublished
Tc-LDH-1	Lactate dehydrogenase		AAB07368	92 aa	Partial sequence	Unpubished
Tc-MUC-1	Mucin-1	TES-120	AAB05820; U39815	176 aa	15.7 kDa Serine-rich peptide backbone with 2 ShKT domains, 39.7 kDa total mass; 120 kDa apparent mobility on SDS-PAGE	Gems and Maizels (1996)
Tc-MUC-2	Mucin-2	TES-120	AF167707	182 aa	16.2 kDa peptide with 2 ShKT domains; 47.8 kDa total.	Loukas et al. (2000b)
Tc-MUC-3	Mucin-3	TES-120	AF167708	269 aa	26.0 kDa threonine-rich peptide with 4 ShKT domains, 45.0 kDa total.	Loukas et al. (2000b)
Tc-MUC-4	Mucin-4	TES-120	AF167709	191 aa	26.0 kDa threonine-rich peptide with 4 ShKT domains, 45.0 kDa total.	Tetteh et al. (1999)
Tc-MUC-5	Mucin-5		AF167710	316 aa	26.0 kDa threonine-rich peptide with 4 ShKT domains, 45.0 kDa total.	Doedens et al. (2001)
Tc-MHC-1	Myosin heavy chain		CAC28360	1814 aa		Obwaller et al. (2001)
Tc-MLC-1	Myosin light chain		U25057	148 aa		Unpublished
Tc-NPA-1	Nematode polyprotein		BAA14015; AAD01628; AAB26196	140 aa	Fragments of longer polyprotein	Christie et al. (1993) and unpublished
Tc-PEB-1	Phosphatidylethanolamine-binding protein	TES-26	P54190, U29761	262 aa	PE-binding domain fused with 2 ShKT domains	Gems et al. (1995)
Tc-PRO-1	Prohibitin		AAB53231	274 aa		Loukas and Maizels (1998)
Tc-SLO-1	Calcium activated channel		ACJ64718	1123 aa		Unpublished
Tc-SOD-1	Cu–Zn superoxide dismutase		AAB00227	190 aa		Unpublished

*Note*: Gene abbreviations follow the convention of two-letter species abbreviation, a three-letter code for gene name (capitalised when referring to a protein product) and a number to distinguish related members of the same gene family.
